# Description of CRISPR/Cas9 development and its prospect in hepatocellular carcinoma treatment

**DOI:** 10.1186/s13046-020-01603-0

**Published:** 2020-06-01

**Authors:** Xiaoling Wu, Weijie Ma, Chengjie Mei, Xi Chen, Ye Yao, Yingyi Liu, Xian Qin, Yufeng Yuan

**Affiliations:** Department of Hepatobiliary and Pancreatic Surgery, Zhongnan Hospital of Wuhan University, Wuhan, 430071 Hubei Province China

**Keywords:** Hepatocellular carcinoma, Clustered regularly interspaced short palindromic repeat/CRISPR-associated nuclease 9, Gene editing, Cancer treatment

## Abstract

Hepatocellular carcinoma (HCC) is one of the most common malignancies today. Patients suffer from HCC since its high malignancy and limited treatment means. With the development of genetic research, new therapeutic strategy comes up in the way of gene editing. Clustered regularly interspaced short palindromic repeat/CRISPR-associated nuclease 9 (CRISPR/Cas9) was discovered as an immune sequence in bacteria and archaea. After artificial transformation and follow-up research, it is widely used as a gene editing tool. In this review, the development of CRISPR/Cas9 is summarized in retrospect. Through the evaluation of novel research in HCC, it is concluded that CRISPR/Cas9 would promote cancer research and provide a new tool for genetic treatment in prospect.

## Background

Liver cancer is one of the most common malignancies in the world, whose global incidence ranks seventh among malignant tumors [1]. The histopathological types of liver cancer mostly are hepatocellular carcinoma and intrahepatic cholangiocarcinoma, of which hepatocellular carcinoma (HCC) accounts for more than 90% of the total cases [2, 3]. It has been confirmed that HCC is closely related to Hepatitis B virus (HBV) infection, hepatitis C virus (HCV) infection, alcoholic cirrhosis and non-alcoholic fatty liver [4, 5]. To date, the treatment of HCC has made little improvement. According to the clinical stage and patient’s own physical condition, surgeons would choose appropriate therapeutic schemes, including liver resection, liver transplantation, radiofrequency ablation, transcatheter arterial chemoembolization (TACE), or take molecular targeting drugs such as sorafenib, levabinib, etc. [6, 7]. Though treatment strategy seems various, the mortality rate of HCC still ranks second among malignancies due to its high recurrent rate and lack of early diagnosis [1]. It is necessary to improve the diagnosis and treatment of HCC in current situation.

In recent years, scientists made great progress on genetic research. More potential oncogenes and antioncogenes were identified in HCC [8]. Diagnosis might also improve in the near future since the reported biomarkers showed better specificity than alpha-fetoprotein (AFP) which is widely used in HCC [9, 10]. At present, different genetic experiments of HCC achieve valuable results in vitro or in vivo [11, 12]. Therefore, genetic therapy presents a cheerful prospect in HCC treatment other than the existing traditional strategies.

Gene editing tool is crucial in cancer research and opens up new ideas for the treatment of HCC since gene knock-out and knock-in are widely used in gene function research. The clustered regularly interspaced short palindromic repeat/CRISPR-associated nuclease 9 (CRISPR/Cas9) system derives from CRISPR family which is a complex immune system existing in microorganisms [13, 14]. After modification, it becomes a versatile technology and gradually replaces former tools to edit gene due to its convenient, low-cost design and broad applications [15].

In this review, the development of CRISPR/Cas9 is summarized based on the analysis of its mechanism, advantages and defects. Besides, we conclude its application in HCC through the assessment of related research in current situation. Learning from some breakthrough of other cancer research, we predict the broadening application of CRISPR/Cas9 in HCC treatment.

## Mechanism of CRISPR/Cas9 technology

**Fig. 1:**
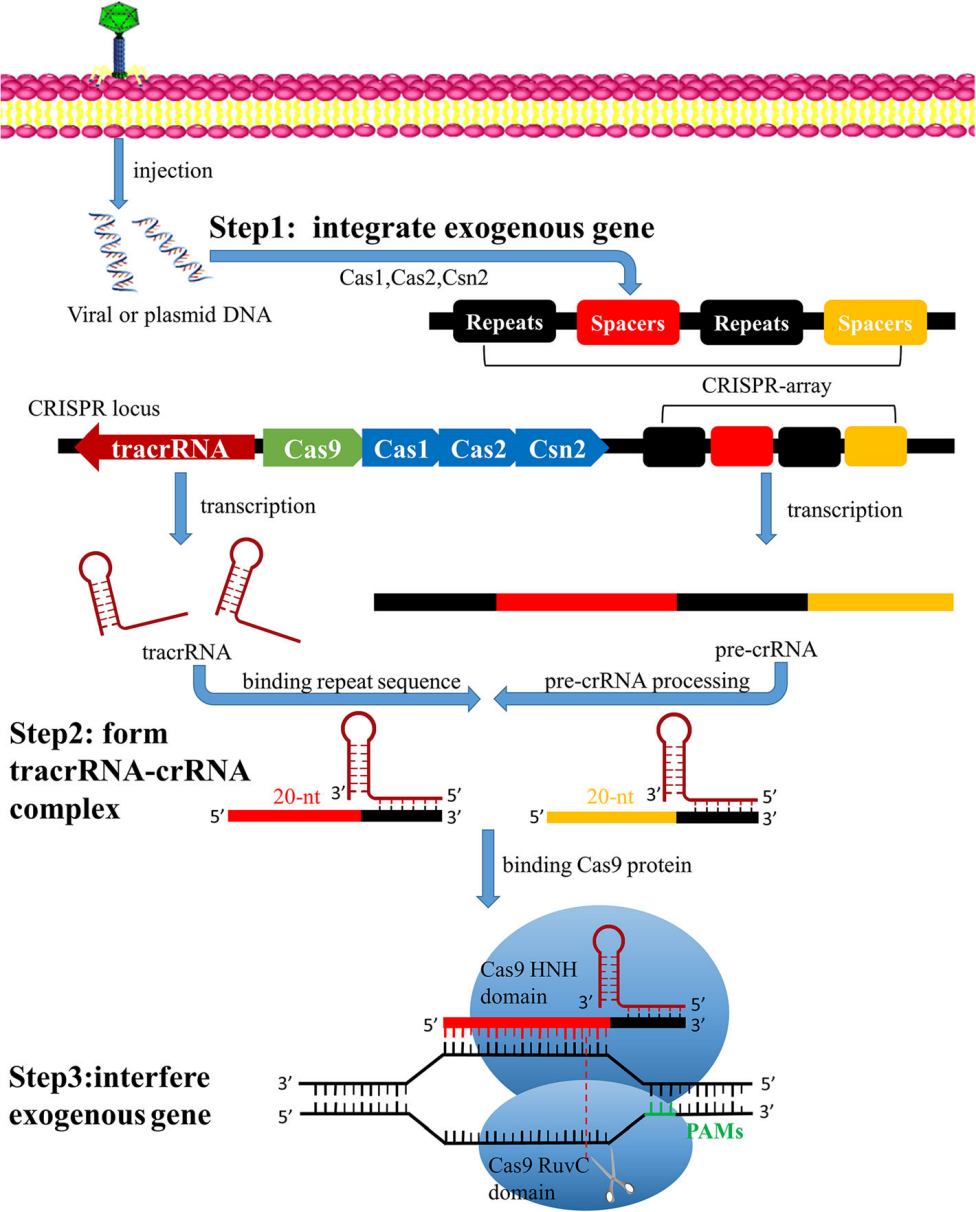
Mechanism of CRISPR/Cas9 gene editing tool. Viral or plasmid DNA would be processed into protospacers and integrated into repeat sequences to form the CRISPR array through Cas1,Cas2 and Csn2. Typical CRISPR locus (from *Streptococcus pyogenes*) consists of tracrRNA sequence, several Cas genes, leader sequence and CRISPR array. CRISPR array transcribes into pre-crRNA. The tracrRNA combines pre-crRNA to form a mature tracrRNA-crRNA complex processed by nucleases. During exogenous gene interference, this complex activates Cas9 endonuclease and recognizes a 20-nt crRNA complementary sequence within exogenous gene, while Cas9 finds PAMs. The double-stranded DNA would be cleaved at 3 nt upstream of the PAMs ultimately by Cas9 endonuclease. Abbreviations: crRNA, CRISPR RNA; tracrRNA, trans-activating crRNA; pre-crRNA, crRNA precursor; PAMs, protospacer adjacent motifs

Figure 1 illustrates the gene editing mechanism of CRISPR/Cas9 through three major steps.

First, exogenous gene should be integrated into CRISPR array.

Once exogenous gene is injected from phage into host, it would be processed into several DNA fragments, or called protospacers. This requires the involvement of Cas1,Cas2 and Csn2 proteins, which are ubiquitous in the CRISPR system [16]. The protospacers would be selected and integrated as new spacers flanked by repeat sequences to form CRISPR array. The selection of protospacers is mostly determined by the short sequences located adjacent to the target sequences within exogenous genome called protospacer adjacent motifs (PAMs) [17]. PAMs are specific to each CRISPR/Cas subtype and recognized as a signal of non-self gene sequence. This integration offers a way to recognize further similar invasion from exogenous gene.

Second, CRISPR locus would produce tracrRNA-crRNA complex.

As shown in Fig. 1, typical CRISPR locus consists of trans-activating CRISPR RNA (tracrRNA) sequence, several Cas genes, leader sequence and CRISPR array. CRISPR system transcribes trans-activating CRISPR RNA (tracrRNA) which is complementary to the repeat sequence from CRISPR array. Meanwhile, CRISPR array transcribes repeats and spacers sequences to produce crRNA precursor (pre-crRNA) complementary to the target sequences within exogenous gene. The pre-crRNA combines tracrRNA, and forms mature tracrRNA-crRNA complex processed by RNase III and other nucleases [18]. This complex would combine with Cas9 protein for later recognition and cleavage of exogenous gene.

Finally, CRISPR/Cas9 system would interfere the invasion of exogenous genome.

Cas9 protein activates nucleases with the combination of tracrRNA and crRNA [15, 19]. When exogenous gene invades again, the Cas9 protein would screen exogenous gene to find specific PAMs as described above. When the PAMs are identified, the 20-nt crRNA would use the spacer sequence to recognize specific target sequence, while double-stranded DNA would be cleaved at 3 nt upstream of the PAMs [20]. Each strand is cleaved by a distinct Cas9 nuclease domain (HNH or RuvC) [21]. The interference protects organism from invasion of exogenous genome and also gives a chance for gene editing since typical CRISPR/Cas9 system brings a break of double-stranded DNA.

For eukaryotes, the damaged gene sequence can be repaired by homology-directed repair (HDR) and non-homologous end joining (NHEJ) after cleavage. NHEJ is the main repairment method for most mammalian cells and tissues. This repairment may cause the insertion or loss of base, which brings frameshift mutation and loss of the gene function, to achieve the aim of knocking out or knocking in target gene [22]. HDR can introduce gene of interest into the genome through the recombination of exogenous DNA donor template with target site [23].

## Brief summary of CRISPR/Cas9 technology development

**Fig. 2:**
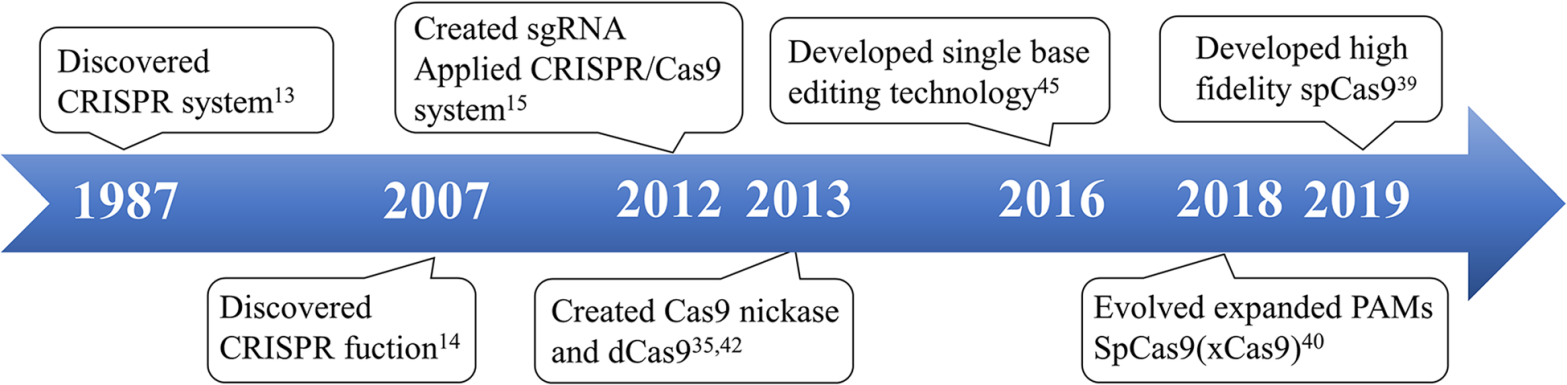
Time shaft of CRISPR/Cas9 development. CRISPR system discovered in 1987 and became gene editing tool in 2012 after the creation of sgRNA. Scientists made several improvements or enhancements in Cas9 technology from different aspects. Abbreviations: sgRNA, single strand RNA; dCas9, dead Cas9 protein;SpCas9, *Streptococcus pyogenes* Cas9

As shown in Fig. 2, CRISPR was first discovered by Japanese scientists in 1987, with its function unknown [13]. In 2007, researchers gradually found that CRISPR was a complex immune system formed by microorganisms themselves to defend invasion of exogenous gene. It integrates exogenous gene fragment into palindromic repeat and generates a RNA-mediated nuclease to cleave exogenous gene [14, 24]. After further research, CRISPR family could be divided into five or six categories by classifying the sequences which encode CRISPR-related proteins [25, 26].

In 2012, CRISPR/Cas9 was employed as a useful gene editing tool (Fig. 2) for the first time. Jinek et al. successfully integrated the double-stranded complex of tracrRNA and crRNA into a single-stranded RNA called single-guide RNA (sgRNA), which could also recognize target gene and activate Cas9 protein to cut double-stranded DNA [15].

Through further research, scientists made several remarkable development in CRISPR/Cas9 technology. Detailed content would be described below.

### Advantages of CRISPR/Cas9

Zinc-finger nucleases (ZFN) and transcription activator-like effector nucleases (TALEN) were widely used as gene editing tools before the artificial transformation of the CRISPR/Cas9 system. Each tool binds the non-specific endonuclease FokI with zinc finger proteins or transcription activator-like effector factors, which could recognize and bind several to tens of specific bases [27, 28].

The modified CRISPR/Cas9 technology shows advantage over both mentioned above, such as the quick, convenient, and low cost of sgRNA construction contrast to the de novo synthesis of guiding protein in ZFN or TALEN. In addition, CRISPR/Cas9 can accomplish multiplex gene editing through construction of multiple sgRNAs targeting different genomic loci [29].

Meanwhile, the efficiency of CRISPR/Cas9 is higher than that of ZFN and TALEN. Ding et al. conducted an experiment to compare the efficiency of CRISPR/Cas9 with that of TALEN. They constructed plasmids containing the sequence of Cas9 protein and transfected into human pluripotent stem cells. Then they designed corresponding TALEN and sgRNA sequences and imported into stem cells by electroporation. Results showed that CRISPR/Cas9 had higher efficiency in mutation of target gene [30].

### Defects of original CRISPR/Cas9

Defects gradually emerge with the use of CRISPR/Cas9 system, the most notable of which is off-target effect. Most researchers believe that the recognition of target gene mainly depends on the guide sequence complementary to 20 nt upstream of PAMs in CRISPR/Cas9 system [31]. However, the designed sgRNA may not fully pair with target sequence within billions of base pairs, followed by off-target effect and low efficiency of gene editing.

As expected, the length of sgRNA is highly correlated with specificity. Since sgRNA contains only 20 complementary nucleotides, non-specific complementary sequence and off target effect is more likely to occur in CRISPR/Cas9 compared with TALEN, whose designed sequence contains 30 to 40 nucleotides [32]. However, it is not optimistic to directly prolong the length of complementary sequence in sgRNA, because it is confirmed that only gene sequence of 14 nucleotides which is composed of 12 nucleotides of sgRNA and 2 nucleotides of PAMs could determine where Cas9 nuclease target for [33]. Further results demonstrated that longer sgRNA and extension of complementary region could only reduce on-target editing efficiency [34, 35]. On the contrary, truncated sgRNA reduced off-target effect without sacrificing gene editing efficiency [36]. Genome-wide homology sequencing is the most straightforward method to examine the presence of non-specific binding with designed sgRNA, but it is not applicable in fundamental research if this technology cannot be simplified due to its defects of time-consuming and high input [37].

In addition, the application of Cas9 protein is also restricted by the recognition of PAMs with specific sequence. For example, *S. pyogenes* Cas9 (SpCas9) must recognize PAMs with NGG nucleotides [38]. Although the repeat frequency of NGG sequence is extremely high in the human genome, it still limits the application of CRISPR/Cas9 [29].

### Improvements of CRISPR/Cas9

**Fig. 3:**
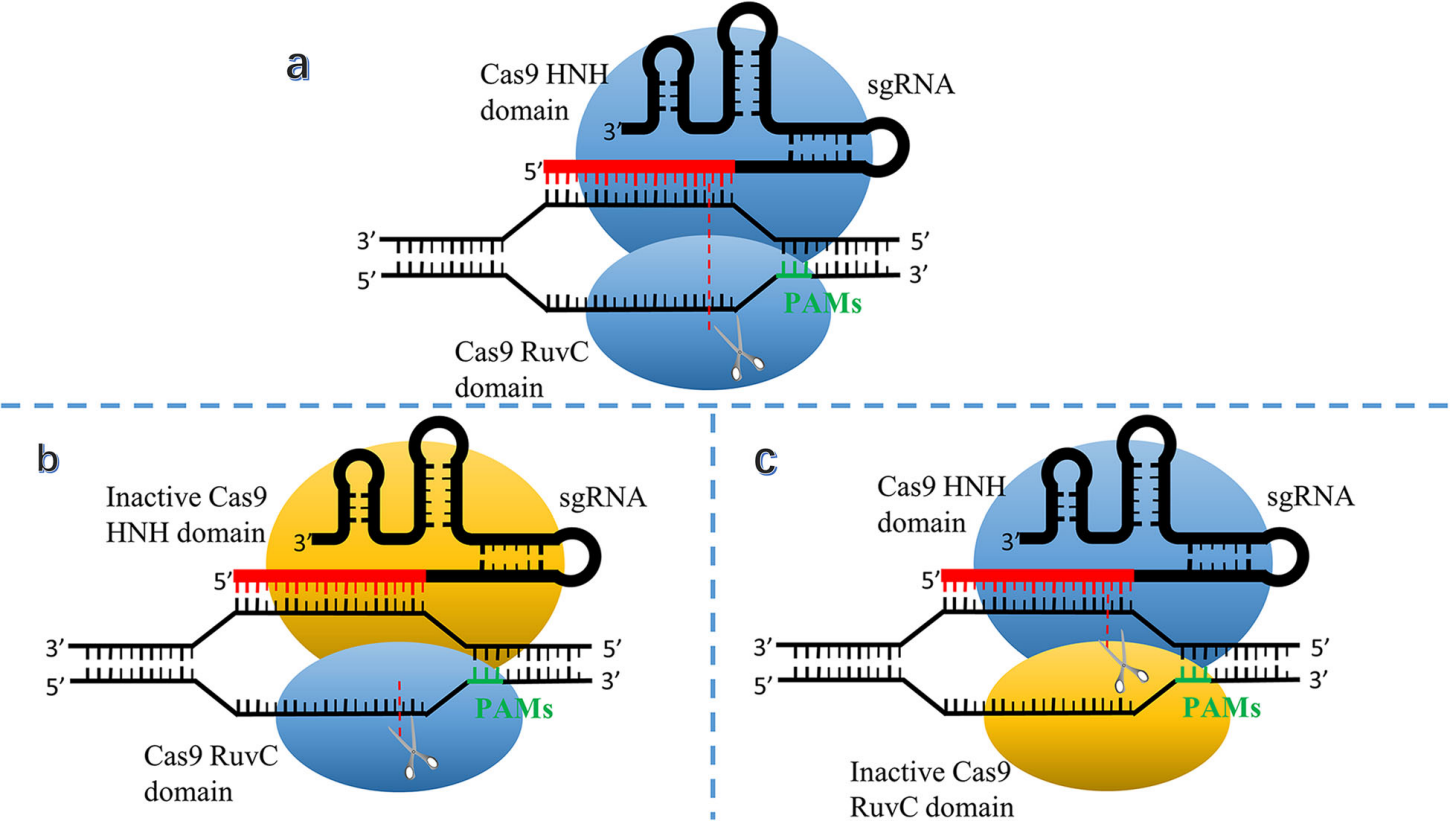
The comparison between typical sgRNA mediated CRISPR/Cas9 and Cas9 nickase. **a** Representative schematic of sgRNA mediated CRISPR/Cas9. The sgRNA derives from tracrRNA-crRNA complex. Each strand is cleaved by a distinct Cas9 nuclease domain (HNH or RuvC). **b**, **c** Representative schematic of Cas9 nickase. One of Cas9 domains inactivation results in single strand cleavage and HDR repairment instead of double strands break. Abbreviations: sgRNA, single strand RNA; PAMs, protospacer adjacent motifs

In response to the dominating defect of off-target effect, scientists made improvement in CRISPR/Cas9 from various aspects. Ran et al. made remarkable achievements in the Cas9 protein mutations in 2013 (mentioned in Fig. 2). They mutated the Cas9 protein domains HNH or RuvC to harvest a Cas9 nickase illustrated in Fig. 3. Under the guidance of sgRNA, the Cas9 nickase cleaves a single strand of DNA, and provides a good repair template for the subsequent HDR process. If an experiment requires cleavage of double-stranded DNA, two designed sgRNA strands could comparatively increase the length of effective complementary sequence and lead to the higher specificity [35].

In 2019, Kleinstiver et al. developed high fidelity SpCas9 (SpCas9-HF1), whose off-target rate cannot be measured by the whole genome sequencing, to perform non-repetitive sequence gene editing in human cell lines (mentioned in Fig. 2). Compared to wild-type SpCas9, it did better in editing 85% of sgRNAs tested in the paper [39].

Another limitation derived from recognition of specific PAMs sequence was overcomed in 2018. Researchers designed xCas9 by modifying SpCas9 (mentioned in Fig. 2), which could recognize PAMs sites of NG, GAA, and GAT, to expand the recognition range of Cas9, and reduce the off-target probability of design products [40].

Scientists also made unremitting efforts to improve efficiency of CRISPR/Cas9. Researchers from Korea discovered the smallest Cas9 protein in Campylobacter jejuni, which consists of 984 amino acids [41]. The smaller protein volume facilitates the construction and transport of vectors.

In summary, designing appropriate sgRNA and selecting suitable CRISPR/Cas9 technology are the best way to improve gene editing efficiency and reduce the mismatch rate.

### Enhanced function of CRISPR/Cas9

**Fig. 4:**
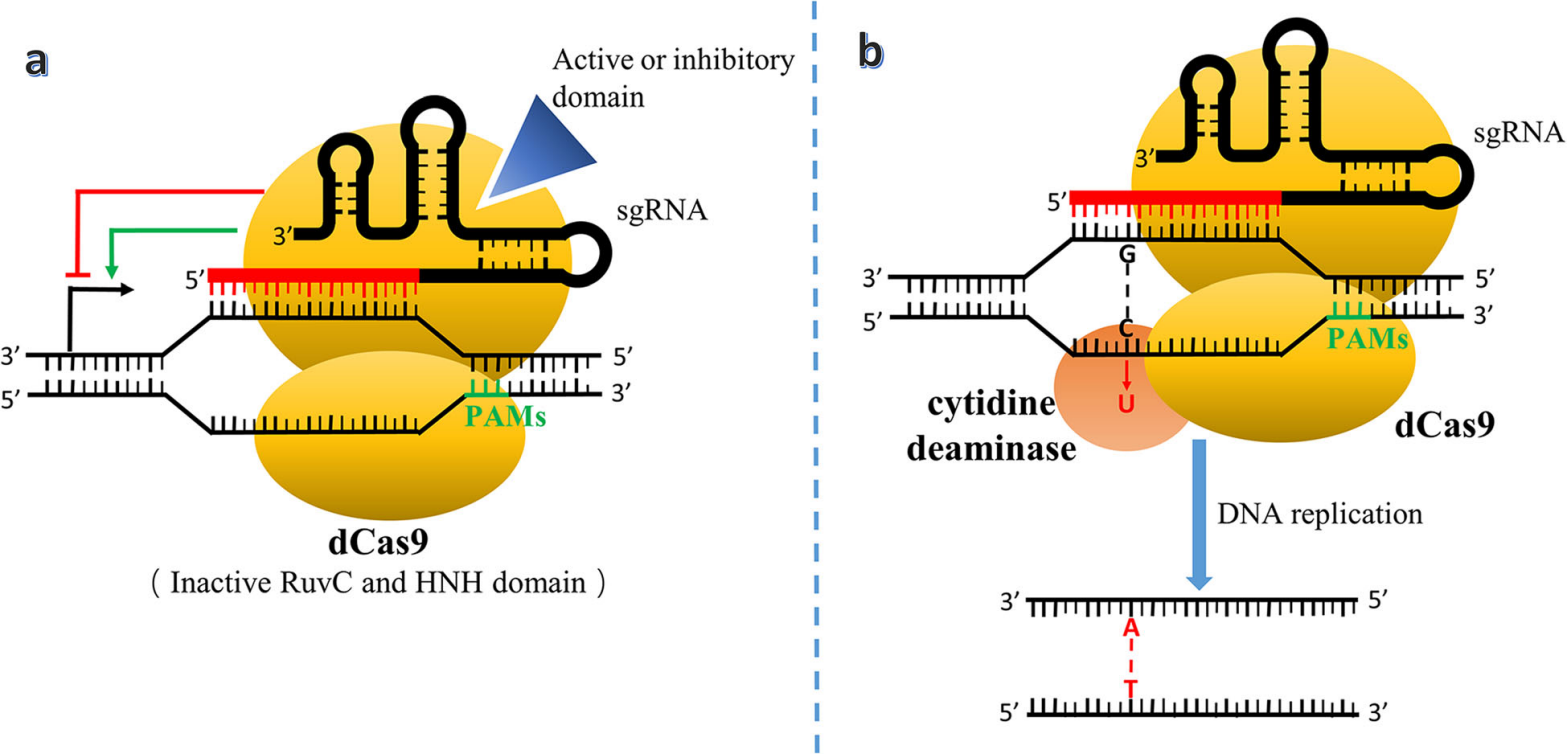
Mechanism of dCas9 and single-base editing technology. **a** Inactive Cas9 domains make dCas9 only recognize and combine with target sequence without break of DNA strand. With the help of active or inhibitory domain, dCas9 increases or decreases the transcription level. **b** The evolution of dCas9 make single base editing possible. Cytidine deaminase exchanges cytosine to uracil and achieves single base editing during DNA replication. Abbreviations: sgRNA, single strand RNA; PAMs, protospacer adjacent motifs; dCas9, dead Cas9 protein

At present, the function of CRISPR/Cas9 technology is far beyond gene knockout. After the modification of the Cas9 protein domain HNH and RuvC, scientists created an inactivated Cas9 protein named dead Cas9 (dCas9) that still specifically recognized the sgRNA binding without nuclease activity in 2013 (mentioned in Fig. 2), [42]. As shown in Fig. 4a, the designed dCas9 could promote or inhibit the transcription of the gene of interest through combination with activating domains such as VP16, VP64, NF-KB etc. or inhibitory domains such as KRAB, MIX1 etc. [43, 44].

In 2016, Komor et al. proposed DNA single-base editing technology, which consisted of a fusion of dCas9 and a cytidine deaminase-encoding gene (Fig. 4b). This complex could catalyze the conversion of cytosine to uracil in the target gene sequence instead by deaminase activity without break of double-stranded DNA, which leads to base substitution without HDR or NHEJ processes during DNA replication or repair [45].

Besides, Perez-Pinera et al. added VP16 to the N-terminus and C-terminus of dCas9 protein and improved the efficiency of multi-gene editing [46]. Bikard et al. constructed and transfected plasmids for editing multiple target genes successfully when studying the drug resistance of the gene aph-3 in *Staphylococcus aureus* [47]. These work provided experimental references for CRISPR/Cas9 to edit different types of cells through one type of medium, or to affect multiple target genes in the same cell simultaneously.

In addition, researchers developed CRISPR/Cas9 tools with various advantages. Perli SD et al. created a self-target guided RNA (stgRNA) that characterizes the time and intensity of gene mutations by the degree of differences between the DNA sequence encoding stgRNA and original sequence [48].

Research team led by Feng Zhang from MIT found a protease Cpf1 in bacteria that protected against viral invasion. It had an endogenous RNase domain that could process pre-crRNA and recognize T-base-rich sequences of PAMs. It performed a lower mismatch rate with shorter sgRNA since it didn’t require tracrRNA coding sequence and showed better edit effect than the Cas9 nickase described above [49, 50]. This discovery indicates that the traditional CRISPR/Cas9 system is not fixed and other CRISPR members might be ignored.

In general, the CRISPR/Cas9 system is constantly improving and new innovation may emerge in CRISPR/Cas9. The enhanced function allows CRISPR/Cas9 to involve in wider range of applications including genetic treatment on cancer.

## Application of CRISPR/Cas9 in the treatment of hepatocellular carcinoma

Carcinoma gene therapy has always been a hot spot of medical research. However, the fact that cancer possesses polygenic and heterogeneous characters reveals the limitation of gene therapy [51]. Besides, carcinoma gene therapy requires high edit efficiency in case that escaped cells still possess malignancy. Compared with the former technology, CRISPR/Cas9 is more frequently used in experiments, given that it can create indels, point mutation, abundant knock-out, abundant knock-in and chromosomal rearrangements [52]. Table 1 briefly summarizes the CRISPR/Cas9 applications or potential use in HCC. Detailed information will be introduced below.

**Table 1:** Overview of CRISPR/Cas9 applications or potential use in HCC treatment

Category	Description of CRISPR/Cas9 function	Reference
Interference in HBV	Edit coding sequence in cccDNA	[53–57]
	Edit conserved region	[53, 58]
	Edit HBsAg protein coding sequence	[59]
	Participate in combination therapy	[60]
Manipulate cancer genome	Knock out or insert specific sequence	[11, 61]
Create hepatocarcinoma mouse model	Knock out specific gene	[62]
	Edit genome in mouse embryonic cells	[63]
Enhance immunotherapy	Modify PD-1 of immune cell	[64, 65]
	Modify CAR-T cell	[66]
	Induce differentiation of iPSCs	[67]
Create potential tool applied in human body	Form delivery agent containing target sgRNA	[68]
Establish CRISPR/Cas9 libraries	Integrate experimental data and prepare for gene screen	[12]

*Abbreviations*: *HBV* hepatitis B virus, *cccDNA* covalently closed circular DNA, *HBsAg* hepatitis B virus surface antigen, *PD*-1 programmed death 1, *CAR-T* chimeric antigen receptor-modified T, *iPSCs* induced pluripotent stem cells, *sgRNA* single strand RNA

### CRISPR/Cas9 technology applications for HBV

Chronic persistent infection of HBV is a major cause of hepatocellular carcinoma [69]. About 30–40% of HBV carriers suffer from hepatocellular carcinoma eventually [70]. At present, there is no antiviral drug that could eliminate HBV [71]. Therefore, gene therapy of HBV would become an effective measure to reduce the number of hepatocellular carcinoma cases.

HBV replication is closely related to covalently closed circular DNA (cccDNA). HBV enters hepatocyte and double-stranded DNA migrates into nucleus to integrate with host chromosome or form cccDNA as transcription template. During the transcription and translation of cccDNA, the core (HBcAg), polymerase, surface (HBsAg) and hepatitis B virus X (HBxAg) protein are generated to produce progeny virus while some transcription production returns to supplement cccDNA [72, 73]. Based on the previous study using TALEN and ZFN, inhibition or elimination of HBV could be achieved with obvious advantages mentioned before by means of CRISPR/Cas9 [74–76].

In 2014, Lin et al. first demonstrated that CRISPR/Cas9 technology could cleave specific sequence in HBV and reduce surface antigen level [53]. Results showed the decrease of HBcAg and HBsAg expression in vitro or in vivo and higher treatment efficiency by multiplex sgRNAs utilization. Other research also confirmed this genetic strategy effective [54–57]. Besides, targeting conserved gene sequence in the HBV genotype could also decrease the replication of HBV, which coincided with other research [58]. Knock-out of HBcAg encoding sequence might eliminate HBV in hepatocytes.

The knockout of HBsAg gene sequence resulted in the decrease of cccDNA copies, HBsAg secretion, tumorigenesis and proliferative ability of HBV-associated hepatocellular carcinoma [59, 77]. On the contrary, HBsAg accumulation would enhance the invasive ability of HBV-associated hepatocellular carcinoma [78].

In 2019, Dash et al. cleared HIV virus successfully in specific experimental group in artificial HIV-infected mouse model [60]. They proposed sequential long-acting slow-effective release antiviral therapy (LASER ART) with the combination of CRISPR/Cas9 gene editing therapy and eliminated HIV in some of the experimental mice. The current situation of HBV treatment, long-term use of antiviral drugs to control viral infection, is similar to treatment of HIV. This study provided new strategy combining antiviral drugs with CRISPR/Cas9 gene therapy for the complete elimination of HBV.

With the deepening research on HBV, more gene sequence will become targets of gene therapy for HBV infection or treatment of hepatocellular carcinoma. CRISPR/Cas9 may also be used in the preparation of vaccines to prevent HBV infection [79]. Nonetheless, efficient delivery of constructed Cas9 plasmids in vivo remains a challenge. Further research should be conducted to to transport Cas9 and sgRNA into target cell of human body and to ensure bio-safety need.

### Manipulation of cancer genome by CRISPR/Cas9

As a versatile tool in gene editing, CRISPR/Cas9 offers scientists a decent choice to manipulate genome of different types of cancer.

Researchers reported that the knockout of hypoxia inducible factor-1α (HIF-1α) by CRISPR/Cas9 improved the therapeutic effect of transarterial embolization (TAE) in cell lines and prolonged the survival of HCC-bearing mice [61]. Based on the evidence that dysregulation of nuclear receptor binding SET domain-containing protein 1 (NSD1) may be related with tumorigenesis in HCC, another paper showed that knockout of NSD1 sequence inhibited proliferation, migration and invasion abilities in HCC cell lines via an inactivation of the Wnt/β-catenin signaling pathway [80].

Besides, scientists employ CRISPR/Cas9 in the therapeutic strategy which inserts target sequence of anti-tumor medicine in HCC to improve drug susceptibility. Chen et al. utilized CRISPR/Cas9 to insert herpes simplex virus type 1 thymidine kinase (HSV1-tk), into the genome of HCC cells. HSV1-tk could phosphorylate ganciclovir and help the synthesis of ganciclovir triphosphate to block genomic replication. HCC cells harboring introduction of HSV1-tk were treated with ganciclovir and mostly went to necrosis in cell culture. In hepatocarcinoma xenografted mice, a reduction of tumor size and mortality was observed [11].

In the future, more and more potential oncogenes would be identified in HCC. More research should be conducted to investigate which oncogene is most suitable for gene therapy and how to avoid the escape of cancer cells.

### Specific construction of hepatocarcinoma mouse model

CRISPR/Cas9 technology is not restricted by different species, and it has been successfully applied in mice, rats, cynomolgus monkeys, domestic pigs, zebrafish and other species [63, 81–84]. For hepatocellular carcinoma, CRISPR/Cas9 technology can help researchers construct mouse models with knockout of target gene, and construct specific gene editing mouse models as well.

In 2014, researchers reported that they used CRISPR/Cas9 to construct a liver cancer mouse model for the first time [62]. Authors transported plasmids containing Cas9 coding sequence and sgRNA targeting the tumor suppressor genes *Pten* and *p53* separately into mice by hydrodynamic tail vein injection and successfully constructed hepatocarcinoma model. On the one hand, this experiment demonstrated hydrodynamic tail vein injection as a method for constructing liver cancer mouse model, and on the other, it created a new experimental scheme to verify specific gene function of liver cancer in animal model [85]. In addition, it is also beneficial to assess potential biosafety hazard in CRISPR/Cas9-mediated treatment of hepatocellular carcinoma by observing growth situation of mice. At present, this method has been used in several cancer research [61, 86, 87].

Hepatocytes can receive gene editing probability up to 40 percentage by hydrodynamic tail vein injection to construct a mouse model of hepatocellular carcinoma [88]. Compared to this method, it would be more thorough to edit mouse embryonic cells when experiments need conventional knockout mice. In 2013, Wang et al. achieved multi-gene knockout of multicellular organisms by injecting Cas9 coding sequence and designed sgRNA into mouse fertilized eggs [63]. Now CRISPR/Cas9 technology is used in embryonic cell engineering. The edited embryonic cells can develop into mature heterozygous animals, whose experimental periods are shorter than those of the ZFN or TALEN-edited embryonic cells. However, compared with the hydrodynamic tail vein injection, this construction method for hepatocellular carcinoma takes more time and costs more. Besides, the volume production is inefficient.

### Enhanced immunotherapy with CRISPR/Cas9

Gene editing of PD-L1 or PD-1 and modification of CAR-T cells become feasible solutions for recent cancer immunotherapy [89, 90]. Immune cells like T cells and B cells express programmed death 1 (PD-1) meanwhile some tumor tissue highly expresses PD-L1. The combination of PD-L1 and PD-1 would disturb autoimmune regulation and weaken tumor killing effect [91, 92]. Chimeric antigen receptor-modified T cells (CAR-T) utilizes specific extracellular single-chain variable fragments (ScFv) to recognize and bind with tumor-associated antigens, thereby recognizing tumor cells and producing immune response [93].

In October 2016, team of Professor You Lu took the lead in the initiation of a tumor treatment trial on the PD-1 gene of immune cell in humans with the help of CRISPR/Cas9 tool. Gene sequence encoding PD-1 of immune cells extracted from metastatic non-small cell lung cancer (NSCLC) patients would be inactivated by CRISPR/Cas9. After expansion in vitro, modified immune cells would be injected back into patients to achieve an enhanced immune response to cancer cells [64]. It offered a template for the similar treatment of HCC. The fundamental study of PD-L1 in hepatocellular carcinoma also achieved relevant results. Guo et al. investigated the effects of chimeric antigen receptor-modified T cells (CAR-T) on the growth of hepatocellular carcinoma and confirmed that CAR-T cells interfered with PD-1 expression have much longer anti-tumor effect. This research indicated that treatment of hepatocellular carcinoma with CAR-T cells could be enhanced by PD-1 modification [65]. It further demonstrated the important role of the PD-L1 and PD-1 in hepatocellular carcinoma treatment.

As for CAR-T therapy in HCC, CRISPR/Cas9 can help CAR-T enhance the immune response to tumors by knocking out genes coding signal molecules or inhibiting immune response receptors. In addition, CRISPR/Cas9 can eliminate the xenografts rejection of T cell by knocking out gene fragments expressing human leukocyte antigen I (HLA I) on T cells. It could make it possible to use universal CAR-T Cells to treat multi-patient and improve utilization efficiency [66].

CRISPR/Cas9 can also process gene engineering in human stem cell or induced pluripotent stem cells (iPSCs). Pluripotent stem cells processed by CRISPR/Cas9 still possess pluripotency through the observation of organ-like formations in cell culture [94]. Therefore patient-derived iPSCs can be modified and induced to differentiation of desired cells in vitro. This theory has been confirmed effective both in iPSCs from *β*-thalassemia patients and DMD patients [95, 96]. In the treatment of HCC, CRISPR/Cas9 can genetically edit human pluripotent stem cells and promote the differentiation of pluripotent stem cells into immune cells such as natural killer cells. Combined with CAR-T gene therapy, it will enhance the immune killing effect on tumor cells after autologous transplantation. Besides, Yin et al. discovered the partial potential of liver cancer stem cells in some CD133-positive cells [67]. CRISPR/Cas9 can also explore gene function by editing target gene in liver cancer stem cells and find ways to reduce the activity of liver cancer stem cells.

### New progress of CRISPR/Cas9 application

The delivery system of CRISPR/Cas9 applied in human body remains a challenge. The development of new materials could solve this problem that the hydrodynamic tail vein injection cannot be applied to human body successfully. Wang et al. compressed the Cas9 sequence and sgRNA plasmids into gold nanoparticles and encapsulated the lipid shell to perform a mature delivery of plasmid, PEG-lipid/AuNPs/Cas9-sgPlk-1 (LACP). This delivery agent could be targeted by the thermoelectric effect of laser and release Cas9 sequence and sgRNA into cell nucleus [68]. Though it was still tested in preclinical stage and delivery efficiency needed assessment, this experiment made it possible to transport CRISPR/Cas9 into target tumor cells in human body.

At present, CRISPR/Cas9 technology expands its application since the construction of sgRNAs libraries. Due to the evolvement of dCas9 described above, CRISPR/Cas9 becomes a versatile tool stronger than RNAi to inhibit or even knockout target sequence [97]. CRISPR/Cas9 libraries have been used to predict specific gene function in tumor proliferation, migration and drug resistance in several aspects [98–100]. Recently, the research team of Renji Hospital in Shanghai proposed a new treatment plan for hepatocellular carcinoma with TP53 mutation. In the initial stage of the study, researchers used sgRNAs sample bank to screen hepatoma cell lines and found that DNA-replication kinase CDC7 acted as an inhibitory site to induce senescence of TP53-mutant hepatoma cell lines selectively. Therefore they induced senescence of hepatoma cell lines with TP53 mutation by inhibiting CDC7. And then they screened for high-throughput compounds to induce apoptosis in senescent liver cancer cells. This “one-two punch” treatment mode illuminates that it is feasible for HCC patients with specific gene mutation to receive drug-targeted treatment [12]. It also demonstrates that the growing of CRISPR/Cas9 technology can improve CRISPR/Cas9 libraries and promote the treatment of hepatocellular carcinoma.

## Conclusion

The CRISPR/Cas9 gene editing technology is favored by researchers because of its remarkable work efficiency, simple construction method and low experimental cost. Accompanied with the extended range of application, CRISPR/Cas9 is more frequently employed in cancer research since the original off-target effect has been weakened during its transformation and innovation. Hepatocellular carcinoma is highly malignant and has limited treatment options. The CRISPR/Cas9 technology provides a new tool for genetic treatment of hepatocellular carcinoma from a different aspect. Although most of gene therapies related with CRISPR/Cas9 still remain in the experimental phase, the novel treatment of hepatocellular carcinoma can be expected with the breakthrough of CRISPR/Cas9 technology.

## Data Availability

Not applicable.

## Abbreviations

CRISPR: Clustered regularly interspaced short palindromic repeat
Cas9: CRISPR-associated nuclease 9
HCC: Hepatocellular carcinoma
HBV: Hepatitis B virus
HCV: Hepatitis C virus
TACE: Transcatheter arterial chemoembolization
AFP: Alpha-fetoprotein
*E. coli*: *Escherichia coli*
PAMs: Protospacer adjacent motifs
tracrRNA: trans-activating CRISPR RNA
pre-crRNA: CRISPR RNA precursor
crRNA: CRISPR RNA
HDR: Homology-directed repair
NHEJ: Non-homologous end joining
sgRNA: single strand RNA
dCas9: dead Cas9 protein
SpCas9: *Streptococcus pyogenes* Cas9
GFP: Green fluorescent protein
ZFN: Zinc-finger nucleases
TALEN: Transcription activator-like effector nucleases
PCR: Polymerase chain reaction
SpCas9-HF1: High fidelity SpCas9
stgRNA: self-target guided RNA
cccDNA: covalently closed circular DNA
HBsAg: Hepatitis B virus surface antigen
PD-1: Programmed death 1
PD-L1: Programmed death 1 ligand 1
CAR-T: Chimeric antigen receptor-modified T
iPSCs: induced pluripotent stem cells
HBcAg: Hepatitis B virus core antigen
HBxAg: Hepatitis B virus X antigen
LASER ART: Long-acting slow-effective release antiviral therapy
HIF-1α: Hypoxia inducible factor-1α
TAE: Transarterial embolization
NSD1: Nuclear receptor binding SET domain-containing protein 1
HSV1-tk: Herpes simplex virus type 1 thymidine kinase
ScFv: Single-chain variable fragments
NSCLC: Non-small cell lung cancer
HLA I: Human leukocyte antigen I

## References

[1] Bray F, Ferlay J, Soerjomataram I, Siegel RL, Torre LA, Jemal A (2018). Global cancer statistics 2018: GLOBOCAN estimates of incidence and mortality worldwide for 36 cancers in 185 countries. CA Cancer J Clin.

[2] Sia D, Villanueva A, Friedman SL, Llovet JM (2017). Liver Cancer cell of origin, molecular class, and effects on patient prognosis. Gastroenterology..

[3] European Society of Gastrointestinal E, European Association for the Study of the Liver (2017). Electronic address eee, European Association for the Study of the L. Role of endoscopy in primary sclerosing cholangitis: European Society of Gastrointestinal Endoscopy (ESGE) and European Association for the Study of the Liver (EASL) clinical guideline. J Hepatol.

[4] Massarweh NN, El-Serag HB (2017). Epidemiology of hepatocellular carcinoma and intrahepatic Cholangiocarcinoma. Cancer Control.

[5] de Martel C, Maucort-Boulch D, Plummer M, Franceschi S (2015). World-wide relative contribution of hepatitis B and C viruses in hepatocellular carcinoma. Hepatology..

[6] Korean Liver Cancer A, National Cancer Center GK (2019). 2018 Korean Liver Cancer association-National Cancer Center Korea practice guidelines for the Management of Hepatocellular Carcinoma. Korean J Radiol.

[7] Kudo M, Finn RS, Qin S, Han KH, Ikeda K, Piscaglia F (2018). Lenvatinib versus sorafenib in first-line treatment of patients with unresectable hepatocellular carcinoma: a randomised phase 3 non-inferiority trial. Lancet..

[8] Ma Y, Luo T, Dong D, Wu X, Wang Y (2018). Characterization of long non-coding RNAs to reveal potential prognostic biomarkers in hepatocellular carcinoma. Gene..

[9] Zhang B, Yang B (1999). Combined alpha fetoprotein testing and ultrasonography as a screening test for primary liver cancer. J Med Screen.

[10] Huang JT, Liu SM, Ma H, Yang Y, Zhang X, Sun H (2016). Systematic review and meta-analysis: circulating miRNAs for diagnosis of hepatocellular carcinoma. J Cell Physiol.

[11] Chen ZH, Yu YP, Zuo ZH, Nelson JB, Michalopoulos GK, Monga S (2017). Targeting genomic rearrangements in tumor cells through Cas9-mediated insertion of a suicide gene. Nat Biotechnol.

[12] Wang C, Vegna S, Jin H, Benedict B, Lieftink C, Ramirez C (2019). Inducing and exploiting vulnerabilities for the treatment of liver cancer. Nature..

[13] Ishino Y, Shinagawa H, Makino K, Amemura M, Nakata A (1987). Nucleotide sequence of the iap gene, responsible for alkaline phosphatase isozyme conversion in Escherichia coli, and identification of the gene product. J Bacteriol.

[14] Barrangou R, Fremaux C, Deveau H, Richards M, Boyaval P, Moineau S (2007). CRISPR provides acquired resistance against viruses in prokaryotes. Science..

[15] Jinek M, Chylinski K, Fonfara I, Hauer M, Doudna JA, Charpentier E (2012). A programmable dual-RNA-guided DNA endonuclease in adaptive bacterial immunity. Science..

[16] Makarova KS, Grishin NV, Shabalina SA, Wolf YI, Koonin EV (2006). A putative RNA-interference-based immune system in prokaryotes: computational analysis of the predicted enzymatic machinery, functional analogies with eukaryotic RNAi, and hypothetical mechanisms of action. Biol Direct.

[17] Mojica FJM, Diez-Villasenor C, Garcia-Martinez J, Almendros C (2009). Short motif sequences determine the targets of the prokaryotic CRISPR defence system. Microbiology..

[18] Marraffini LA, Sontheimer EJ (2010). CRISPR interference: RNA-directed adaptive immunity in bacteria and archaea. Nat Rev Genet.

[19] Chylinski K, Le Rhun A, Charpentier E (2013). The tracrRNA and Cas9 families of type II CRISPR-Cas immunity systems. RNA Biol.

[20] Chen H, Choi J, Bailey S (2014). Cut site selection by the two nuclease domains of the Cas9 RNA-guided endonuclease. J Biol Chem.

[21] Garneau JE, Dupuis ME, Villion M, Romero DA, Barrangou R, Boyaval P (2010). The CRISPR/Cas bacterial immune system cleaves bacteriophage and plasmid DNA. Nature..

[22] Jiang F, Doudna JA (2017). CRISPR-Cas9 structures and mechanisms. Annu Rev Biophys.

[23] Zhang S, Guo F, Yan W, Dai Z, Dong W, Zhou J (2019). Recent advances of CRISPR/Cas9-based genetic engineering and transcriptional regulation in industrial biology. Front Bioeng Biotechnol.

[24] Walsh RM, Hochedlinger K (2013). A variant CRISPR-Cas9 system adds versatility to genome engineering. Proc Natl Acad Sci U S A.

[25] Chylinski K, Makarova KS, Charpentier E, Koonin EV (2014). Classification and evolution of type II CRISPR-Cas systems. Nucleic Acids Res.

[26] Wright AV, Nunez JK, Doudna JA (2016). Biology and applications of CRISPR systems: harnessing Nature's toolbox for genome engineering. Cell..

[27] Kim YG, Cha J, Chandrasegaran S (1996). Hybrid restriction enzymes: zinc finger fusions to Fok I cleavage domain. Proc Natl Acad Sci U S A.

[28] Boch J, Scholze H, Schornack S, Landgraf A, Hahn S, Kay S (2009). Breaking the code of DNA binding specificity of TAL-type III effectors. Science..

[29] Cong L, Ran FA, Cox D, Lin S, Barretto R, Habib N (2013). Multiplex genome engineering using CRISPR/Cas systems. Science..

[30] Ding Q, Regan SN, Xia Y, Oostrom LA, Cowan CA, Musunuru K (2013). Enhanced efficiency of human pluripotent stem cell genome editing through replacing TALENs with CRISPRs. Cell Stem Cell.

[31] Kleinstiver BP, Prew MS, Tsai SQ, Topkar VV, Nguyen NT, Zheng Z (2015). Engineered CRISPR-Cas9 nucleases with altered PAM specificities. Nature..

[32] Juillerat A, Dubois G, Valton J, Thomas S, Stella S, Marechal A (2014). Comprehensive analysis of the specificity of transcription activator-like effector nucleases. Nucleic Acids Res.

[33] Larson MH, Gilbert LA, Wang X, Lim WA, Weissman JS, Qi LS (2013). CRISPR interference (CRISPRi) for sequence-specific control of gene expression. Nat Protoc.

[34] Cho SW, Kim S, Kim Y, Kweon J, Kim HS, Bae S (2014). Analysis of off-target effects of CRISPR/Cas-derived RNA-guided endonucleases and nickases. Genome Res.

[35] Ran FA, Hsu PD, Lin CY, Gootenberg JS, Konermann S, Trevino AE (2013). Double nicking by RNA-guided CRISPR Cas9 for enhanced genome editing specificity. Cell..

[36] Fu Y, Sander JD, Reyon D, Cascio VM, Joung JK (2014). Improving CRISPR-Cas nuclease specificity using truncated guide RNAs. Nat Biotechnol.

[37] Wiles MV, Qin W, Cheng AW, Wang H (2015). CRISPR-Cas9-mediated genome editing and guide RNA design. Mamm Genome.

[38] Jiang W, Bikard D, Cox D, Zhang F, Marraffini LA (2013). RNA-guided editing of bacterial genomes using CRISPR-Cas systems. Nat Biotechnol.

[39] Loureiro A, da Silva GJ. CRISPR-Cas: Converting A Bacterial Defence Mechanism into A State-of-the-Art Genetic Manipulation Tool. Antibiotics. 2019;8(1). 10.3390/antibiotics8010018 PMC6466564 30823430

[40] Hu JH, Miller SM, Geurts MH, Tang W, Chen L, Sun N (2018). Evolved Cas9 variants with broad PAM compatibility and high DNA specificity. Nature..

[41] Kim E, Koo T, Park SW, Kim D, Kim K, Cho HY (2017). In vivo genome editing with a small Cas9 orthologue derived from campylobacter jejuni. Nat Commun.

[42] Bikard D, Jiang W, Samai P, Hochschild A, Zhang F, Marraffini LA (2013). Programmable repression and activation of bacterial gene expression using an engineered CRISPR-Cas system. Nucleic Acids Res.

[43] Perez-Pinera P, Kocak DD, Vockley CM, Adler AF, Kabadi AM, Polstein LR (2013). RNA-guided gene activation by CRISPR-Cas9-based transcription factors. Nat Methods.

[44] Ji W, Lee D, Wong E, Dadlani P, Dinh D, Huang V (2014). Specific gene repression by CRISPRi system transferred through bacterial conjugation. ACS Synth Biol.

[45] Komor AC, Kim YB, Packer MS, Zuris JA, Liu DR (2016). Programmable editing of a target base in genomic DNA without double-stranded DNA cleavage. Nature..

[46] Hsu PD, Lander ES, Zhang F (2014). Development and applications of CRISPR-Cas9 for genome engineering. Cell..

[47] Bikard D, Euler CW, Jiang W, Nussenzweig PM, Goldberg GW, Duportet X (2014). Exploiting CRISPR-Cas nucleases to produce sequence-specific antimicrobials. Nat Biotechnol.

[48] Perli SD, Cui CH, Lu TK. Continuous genetic recording with self-targeting CRISPR-Cas in human cells. Science. 2016;353(6304). 10.1126/science.aag0511 27540006

[49] Zetsche B, Gootenberg JS, Abudayyeh OO, Slaymaker IM, Makarova KS, Essletzbichler P (2015). Cpf1 is a single RNA-guided endonuclease of a class 2 CRISPR-Cas system. Cell..

[50] Kim D, Kim J, Hur JK, Been KW, Yoon SH, Kim JS (2016). Genome-wide analysis reveals specificities of Cpf1 endonucleases in human cells. Nat Biotechnol.

[51] Xiao-Jie L, Hui-Ying X, Zun-Ping K, Jin-Lian C, Li-Juan J (2015). CRISPR-Cas9: a new and promising player in gene therapy. J Med Genet.

[52] Baliou S, Adamaki M, Kyriakopoulos AM, Spandidos DA, Panayiotidis M, Christodoulou I (2018). CRISPR therapeutic tools for complex genetic disorders and cancer (review). Int J Oncol.

[53] Lin SR, Yang HC, Kuo YT, Liu CJ, Yang TY, Sung KC (2014). The CRISPR/Cas9 system facilitates clearance of the intrahepatic HBV templates in vivo. Mol Ther Nucleic Acids.

[54] Kennedy EM, Kornepati AV, Cullen BR (2015). Targeting hepatitis B virus cccDNA using CRISPR/Cas9. Antivir Res.

[55] Seeger C, Sohn JA (2016). Complete Spectrum of CRISPR/Cas9-induced mutations on HBV cccDNA. Mol Ther.

[56] Ramanan V, Shlomai A, Cox DB, Schwartz RE, Michailidis E, Bhatta A (2015). CRISPR/Cas9 cleavage of viral DNA efficiently suppresses hepatitis B virus. Sci Rep.

[57] Dong C, Qu L, Wang H, Wei L, Dong Y, Xiong S (2015). Targeting hepatitis B virus cccDNA by CRISPR/Cas9 nuclease efficiently inhibits viral replication. Antivir Res.

[58] Liu X, Hao R, Chen S, Guo D, Chen Y (2015). Inhibition of hepatitis B virus by the CRISPR/Cas9 system via targeting the conserved regions of the viral genome. J Gen Virol.

[59] Zhen S, Hua L, Liu YH, Gao LC, Fu J, Wan DY (2015). Harnessing the clustered regularly interspaced short palindromic repeat (CRISPR)/CRISPR-associated Cas9 system to disrupt the hepatitis B virus. Gene Ther.

[60] Dash PK, Kaminski R, Bella R, Su H, Mathews S, Ahooyi TM (2019). Sequential LASER ART and CRISPR treatments eliminate HIV-1 in a subset of infected humanized mice. Nat Commun.

[61] Liu Q, Fan D, Adah D, Wu Z, Liu R, Yan QT (2018). CRISPR/Cas9mediated hypoxia inducible factor1alpha knockout enhances the antitumor effect of transarterial embolization in hepatocellular carcinoma. Oncol Rep.

[62] Xue W, Chen S, Yin H, Tammela T, Papagiannakopoulos T, Joshi NS (2014). CRISPR-mediated direct mutation of cancer genes in the mouse liver. Nature..

[63] Wang H, Yang H, Shivalila CS, Dawlaty MM, Cheng AW, Zhang F (2013). One-step generation of mice carrying mutations in multiple genes by CRISPR/Cas-mediated genome engineering. Cell..

[64] Cyranoski D (2016). CRISPR gene-editing tested in a person for the first time. Nature..

[65] Guo X, Jiang H, Shi B, Zhou M, Zhang H, Shi Z (2018). Disruption of PD-1 enhanced the anti-tumor activity of chimeric antigen receptor T cells against hepatocellular carcinoma. Front Pharmacol.

[66] Liu X, Zhang Y, Cheng C, Cheng AW, Zhang X, Li N (2017). CRISPR-Cas9-mediated multiplex gene editing in CAR-T cells. Cell Res.

[67] Yin S, Li J, Hu C, Chen X, Yao M, Yan M (2007). CD133 positive hepatocellular carcinoma cells possess high capacity for tumorigenicity. Int J Cancer.

[68] Wang P, Zhang L, Zheng W, Cong L, Guo Z, Xie Y (2018). Thermo-triggered release of CRISPR-Cas9 system by lipid-encapsulated gold nanoparticles for tumor therapy. Angew Chem.

[69] Lok AS (2004). Prevention of hepatitis B virus-related hepatocellular carcinoma. Gastroenterology..

[70] Fares N, Peron JM (2013). Epidemiology, natural history, and risk factors of hepatocellular carcinoma. La Revue du praticien.

[71] Trepo C, Chan HL, Lok A (2014). Hepatitis B virus infection. Lancet..

[72] Guo JT, Guo H (2015). Metabolism and function of hepatitis B virus cccDNA: implications for the development of cccDNA-targeting antiviral therapeutics. Antivir Res.

[73] Lin G, Zhang K, Li J (2015). Application of CRISPR/Cas9 technology to HBV. Int J Mol Sci.

[74] Chen J, Zhang W, Lin J, Wang F, Wu M, Chen C (2014). An efficient antiviral strategy for targeting hepatitis B virus genome using transcription activator-like effector nucleases. Mol Ther.

[75] Bloom K, Ely A, Mussolino C, Cathomen T, Arbuthnot P (2013). Inactivation of hepatitis B virus replication in cultured cells and in vivo with engineered transcription activator-like effector nucleases. Mol Ther.

[76] Weber ND, Stone D, Sedlak RH, De Silva Feelixge HS, Roychoudhury P, Schiffer JT (2014). AAV-mediated delivery of zinc finger nucleases targeting hepatitis B virus inhibits active replication. PLoS One.

[77] Song J, Zhang X, Ge Q, Yuan C, Chu L, Liang HF (2018). CRISPR/Cas9-mediated knockout of HBsAg inhibits proliferation and tumorigenicity of HBV-positive hepatocellular carcinoma cells. J Cell Biochem.

[78] Cheng S, Zhang B, Du JY, Jin YH, Lang HY, Zeng LH (2017). Hepatitis B surface antigen promotes the invasion of hepatitis B virus-related hepatocellular carcinoma cells by Upregulation of toll-like receptor 2. Viral Immunol.

[79] Okoli A, Okeke MI, Tryland M, Moens U. CRISPR/Cas9-Advancing Orthopoxvirus Genome Editing for Vaccine and Vector Development. Viruses. 2018;10(1). 10.3390/v10010050 PMC5795463 29361752

[80] Zhang S, Zhang F, Chen Q, Wan C, Xiong J, Xu J (2019). CRISPR/Cas9-mediated knockout of NSD1 suppresses the hepatocellular carcinoma development via the NSD1/H3/Wnt10b signaling pathway. J Exp Clin Cancer Res.

[81] Zhou X, Xin J, Fan N, Zou Q, Huang J, Ouyang Z (2015). Generation of CRISPR/Cas9-mediated gene-targeted pigs via somatic cell nuclear transfer. Cell Mol Life Sci.

[82] Niu Y, Shen B, Cui Y, Chen Y, Wang J, Wang L (2014). Generation of gene-modified cynomolgus monkey via Cas9/RNA-mediated gene targeting in one-cell embryos. Cell..

[83] Ma Y, Chen W, Zhang X, Yu L, Dong W, Pan S (2016). Increasing the efficiency of CRISPR/Cas9-mediated precise genome editing in rats by inhibiting NHEJ and using Cas9 protein. RNA Biol.

[84] Hruscha A, Krawitz P, Rechenberg A, Heinrich V, Hecht J, Haass C (2013). Efficient CRISPR/Cas9 genome editing with low off-target effects in zebrafish. Development..

[85] Zhang G, Budker V, Wolff JA (1999). High levels of foreign gene expression in hepatocytes after tail vein injections of naked plasmid DNA. Hum Gene Ther.

[86] Weber J, Ollinger R, Friedrich M, Ehmer U, Barenboim M, Steiger K (2015). CRISPR/Cas9 somatic multiplex-mutagenesis for high-throughput functional cancer genomics in mice. Proc Natl Acad Sci U S A.

[87] Song CQ, Li Y, Mou H, Moore J, Park A, Pomyen Y (2017). Genome-wide CRISPR screen identifies regulators of mitogen-activated protein kinase as suppressors of Liver tumors in mice. Gastroenterology..

[88] Pankowicz FP, Jarrett KE, Lagor WR, Bissig KD (2017). CRISPR/Cas9: at the cutting edge of hepatology. Gut..

[89] Iwai Y, Hamanishi J, Chamoto K, Honjo T (2017). Cancer immunotherapies targeting the PD-1 signaling pathway. J Biomed Sci.

[90] Reghupaty SC, Sarkar D (2019). Current Status of Gene Therapy in Hepatocellular Carcinoma. Cancers.

[91] Iwai Y, Ishida M, Tanaka Y, Okazaki T, Honjo T, Minato N (2002). Involvement of PD-L1 on tumor cells in the escape from host immune system and tumor immunotherapy by PD-L1 blockade. Proc Natl Acad Sci U S A.

[92] Okazaki T, Honjo T (2006). The PD-1-PD-L pathway in immunological tolerance. Trends Immunol.

[93] Wang Z, Wu Z, Liu Y, Han W (2017). New development in CAR-T cell therapy. J Hematol Oncol.

[94] Schwank G, Koo BK, Sasselli V, Dekkers JF, Heo I, Demircan T (2013). Functional repair of CFTR by CRISPR/Cas9 in intestinal stem cell organoids of cystic fibrosis patients. Cell Stem Cell.

[95] Xie F, Ye L, Chang JC, Beyer AI, Wang J, Muench MO (2014). Seamless gene correction of beta-thalassemia mutations in patient-specific iPSCs using CRISPR/Cas9 and piggyBac. Genome Res.

[96] Li HL, Fujimoto N, Sasakawa N, Shirai S, Ohkame T, Sakuma T (2015). Precise correction of the dystrophin gene in duchenne muscular dystrophy patient induced pluripotent stem cells by TALEN and CRISPR-Cas9. Stem Cell Rep.

[97] Evers B, Jastrzebski K, Heijmans JP, Grernrum W, Beijersbergen RL, Bernards R (2016). CRISPR knockout screening outperforms shRNA and CRISPRi in identifying essential genes. Nat Biotechnol.

[98] Chen S, Sanjana NE, Zheng K, Shalem O, Lee K, Shi X (2015). Genome-wide CRISPR screen in a mouse model of tumor growth and metastasis. Cell..

[99] Zhou Y, Zhu S, Cai C, Yuan P, Li C, Huang Y (2014). High-throughput screening of a CRISPR/Cas9 library for functional genomics in human cells. Nature..

[100] Wei L, Lee D, Law CT, Zhang MS, Shen J, Chin DW (2019). Genome-wide CRISPR/Cas9 library screening identified PHGDH as a critical driver for Sorafenib resistance in HCC. Nat Commun.

